# Tension induces intervertebral disc degeneration via endoplasmic reticulum stress-mediated autophagy

**DOI:** 10.1042/BSR20190578

**Published:** 2019-08-07

**Authors:** Jiangwei Chen, Zunwen Lin, Kui Deng, Bin Shao, Dong Yang

**Affiliations:** 1 Department of Orthopedics, The First Affiliated Hospital of Nanchang University, Nanchang, China; 2 Department of Orthopedics, Jingdezhen First People’s Hospital, Jingdezhen, China

**Keywords:** autophagy, cyclic deformation, endoplasmic reticulum stress, intervertebral disc degeneration

## Abstract

Background: Intervertebral disc degeneration is a common degenerative disease. The present study aimed to explore the role and mechanism of tension-induced endoplasmic reticulum stress in intervertebral disc degeneration.

Methods: Intervertebral disc degeneration models of SD rat were analyzed for apoptosis, the expression of Poly(ADP-ribose) polymerase (PARP), Caspase-12, Caspase-3, LC3, Beclin-1 and CHOP using immunohistochemistry, qPCR and Western blot analysis. Annulus fibrosus cells of intervertebral disc were isolated, subjected to cyclic deformation stress and analyzed for ROS and apoptosis, lysosome activity and expression of genes. The cells were knockdown with siRNA or treated with endoplasmic reticulum stress inhibitor 4-PBA and assayed for ROS, apoptosis, lysosome activity and gene expression.

Results: Compared with the controls, intervertebral disc degeneration was observed through X-rays examinations and HS staining. Apoptosis and expression of PARP, Caspase-12, Caspase-3, LC3, Beclin-1 and CHOP were significantly increased in the intervertebral disc tissue of the models. In mechanic mimic experiments, the primary annulus fibrosus cells were subjected to 18% cyclic deformation, ROS and apoptosis as well as the activity of lysosome were increased. Similarly, the expression of PARP, Caspase-12, Caspase-3, LC3, Beclin-1 and CHOP was also increased significantly after deformation treatment. On other hand, when the cells were treated with 9 mM 4-PBA and/or CHOP-siRNA4, the apoptosis rate, ROS level, lysosome activity and expression of PARP, Caspase-12, Caspase-3, LC3, Beclin-1 and CHOP were significantly reduced.

Conclusions: Autophagy reaction mediated by endoplasmic reticulum stress plays important rale in tension-induced intervertebral disc degeneration. Intervertebral disc degeneration likely results from interactions between autophagy, apoptosis and reticulum stress, and is ROS-dependent.

## Background

Intervertebral disc degeneration is a common degenerative disease, which often results in neck and shoulder pain and seriously affects the quality of life of the patients. At present, the clinical treatment of this disease is relied on operation to relieve nerve compression. It is still not possible to fundamentally prevent or reverse the occurrence and development of intervertebral disc degeneration. Studies have shown that the degeneration of intervertebral disc tissue is closely related to the apoptosis of disc cells and metabolic imbalance of extracellular proteoglycan and type II collagen [1,2] and degenerated intervertebral disc tissues often have a large number of apoptotic cells [3,4]. Autophagy, is also involved in the development of many degenerative diseases, such as Parkinson’s disease, Alzheimer’s disease, osteoarthritis and rheumatoid arthritis [5–7]. Autophagy is a double-edged sword: on the one hand, moderate levels of autophagy can provide sufficient metabolic substrates and energy to ensure or maintain cell metabolism and promote cell survival; on the other hand, excessive levels of autophagy can deplete resources and lead to cell death [8]. There is an interaction between autophagy and apoptotic signals. Apoptosis repressors Bcl-2 and Bcl-XL may inhibit apoptosis by reducing the permeability of mitochondrial membrane and autophagy by binding to autophagy protein Beclin-1, suggesting that Bcl-2 and Bcl-XL can inhibit both autophagy and apoptosis. However, a large number of studies have shown that activation of autophagy can protect cells and inhibit apoptosis [9]. Endoplasmic reticulum is an important subcellular organelle that regulates intracellular calcium homeostasis, protein synthesis and apoptosis. The role of endoplasmic reticulum stress is to restore the homeostasis in the endoplasmic reticulum and protect cells. However, when the stress is too strong or persistent, the endoplasmic reticulum dysfunction may not be restored fully, and the cells will enter the apoptotic pathway [10]. Endoplasmic reticulum stress can not only affect cell survival or death through autophagy, but also directly induce cell death [11]. Our previous works investigated the autophagy and apoptosis levels in intervertebral disc cells under nutrition deprivation and oxidative stress conditions [12–14]. Compared with these biological factors, mechanical factors may be more critical in the process of intervertebral disc degeneration. It is found that the incidence of degenerative diseases of the manual workers is higher, which may be due to the frequent flexion and extension, leading to excessive stress stimulation of the intervertebral disc, and accelerated degeneration [15]. The mechanism underlying apoptosis of intervertebral disc cells induced by cyclic tension, a mimic of frequent flexion and extension in manual workers is largely unclear. A better understanding of ROS-dependent endoplasmic reticulum stress-autophagy reaction may provide insight on the regulation of autophagy level or upstream endoplasmic reticulum stress level, and help develop strategy to reduce the apoptotic level in annulus fibrosus cells, thus delaying the occurrence of degeneration of intervertebral disc tissue.

In the present study, we investigated the role of autophagy in tension-induced disc degeneration. The findings would provide better understanding of mechanism underlying the degeneration and clues for the prevention and treatment of the disease.

## Materials and methods

### Experimental animals

Twenty healthy male SD rats purchased from Slarc Laboratory Animals, Shanghai, China (permit no. SCXK (Shanghai) 2013-0016) were used for the present study. Five animals were used in each group, and all animal experiments were conducted at the First Affiliated Hospital of Nanchang University and the experimental protocols were approved by The Research Ethnic Committee of the First Affiliated Hospital of Nanchang University (approval number: 2019-011).

### Reagents and instruments

TRIzo1 reagent (CW0580S), ultrapure RNA extraction kit (CW0581M) and HiFiScript first strand cDNA synthesis kit (CW2569M) were purchased from CWBIO, Shanghai, China; TUNEL kit (C1088) was purchased from Keygen, Shanghai, China; rabbit polyclonal antibody against LC3 (bs-8878R, 1/200), mouse monoclonal antibody against Beclin (bs-3315M,1/600) and rabbit polyclonal antibody against Caspase-3 (bs-0081R,1/200) were purchased from Bioss, Beijing, China; rabbit monoclonal antibody against PARP (ab32138, 1/2000) was purchased from Abcam, U.S.A.; rabbit polyclonal antibodies against Caspase-12 (A0556), CHOP (A0221, 1/800), GAPDH (TA-08, 1/2000)), goat anti-mouse IgG (ZB-2305, 1/2000) and goat anti-rabbit IgG (ZB-2301, 1/2000) were purchased from Boster, Wuhan, China; 4-PBA was obtained from MCE, U.S.A.). Tension systems (FX-4000T) was purchased from Flexcellint, U.S.A. Fluorescence microscopy (742BR1154), ultrasensitive chemiluminescence imaging system (ChemiDoc™ XRS+) and fluorescent PCR instrument were purchased from Bio-Rad, U.S.A. Flow cytometer (NovoCyte) was a product of Eisen Biologicals, Hangzhou, China.

### Lumbar intervertebral disc degeneration models

SD rats were anesthetized with 1% pentobarbital sodium at the dosage of 45 mg/kg. The articular processes at lumbar vertebrae between L1 and L6 and thoracic vertebrae between T11 and T13 were cut and sutured to generate the degeneration models. For sham operation, an incision along the spine was made but the processes were not cut. Normal rats were used as control.

### Hematoxylin and Eosin staining

Hematoxylin and Eosin (HE) staining was performed as previously described [16].

### Immunohistochemistry

A total of 20 μm transverse sections of tissue were incubated with antibodies for 2 h at room temperature followed by incubation with appropriate secondary antibodies for 1 h, and mounted with ProLong antifade reagent (Invitrogen). Each antibody series was performed on all tissue sections simultaneously to control for variations in processing and allow quantitative comparisons of staining intensity. Images for a single antibody series were acquired at the same fluorescence intensity to keep imaging conditions constant and allow quantitative comparisons of staining intensity.

### TUNEL assay

TUNEL assays were conducted as previously reported [17] and stained slides were viewed under fluorescence microscope.

### Real-time quantitative PCR for gene expression

Total RNA isolated using the TRIzo1 reagent and reverse transcription was performed with 200 ng of RNA in a total volume of 10 μl using the HiFiScript first strand cDNA synthesis kit according to manufacturer’s recommendations. RT-qPCR was performed on the 7900HT Fast Real-Time PCR system using TaqMan gene expression assays probes (Applied Biosystems). Rat glyceraldehyde-3-phosphate dehydrogenase, *GADPH*, was used as an internal control. The PCR was carried out in a total volume of 10 μl containing 1.5 μl of diluted and pre-amplified cDNA, 10 μl of TaqMan Gene Expression Master Mix and 1 μl of each fluorescence TaqMan probe using the primers listed in Table 1. The cycling conditions were 50°C for 2 min, 95°C for 10 min followed by 40 cycles, each one consisting of 15 s at 95°C and 1 min at 60°C. Samples were run in triplicate and the mean value was calculated for each case.

**Table 1:** Primers for qPCR

Primer	Sequence
LC3 F	ATGGCGGCGTCTTTGTG
LC3 R	TGGATTTCTTCAGTTGCTTGG
Beclin1 F	CAGCGGCATTACCTACAAAA
Beclin1 R	TTCTCCTGAAGCCTCCTCC
CHOP F	ATGTGACGAGGTGGATGGA
CHOP R	TTTCGGCTGGGATTCTGT
PARP F	ATGTGACGAGGTGGATGGA
PARP R	TTTCGGCTGGGATTCTGT
Caspase-12 F	CATTCCTGGTCTTTATGTCCC
Caspase-12 R	GGCTATCCCTTTGCTTGTG
Caspase3 F	AAAGCCGAAACTCTTCATCA
Caspase3 R	GTCTCAATACCGCAGTCCAG
GAPDH F	TACCCACGGCAAGTTCAA
GAPDH R	ACCAGCATCACCCCATTT

F: forward primer; R: reverse primer

The data were managed using the Applied Biosystems software RQ Manager v1.2.1. Relative expression was calculated by using comparative *C*_t_ method and obtaining the fold change value (2^−ΔΔ*C*^_t_) according to previously described protocol [18].

### Western blot analysis

After different treatments, the cells were lysed with RIPA buffer that contains protease and phosphotase inhibitors cocktail and quantitated using BAC kit according to manufacturer’s instructions. Approximately 50 μg protein was applied to 12% polyacrylamide gel electrophoresis (SDS/PAGE), transferred to a PVDF membrane, and then detected by the proper primary and secondary antibodies before visualization with a chemiluminescence kit. The intensity of blot signals was quantitated using ultrasensitive chemiluminescence imaging system (ChemiDocXRS+).

### Detection of apoptosis by flow cytometry

Cells were pretreated with MTE for 12 h and then co-treated with or without gefitinib for another 72 h. Floating and adherent cells were collected and suspended in PBS, labeled with Annexin V and propidiumiodide (PI) following the manufacturer’s instructions (Biosea Biotechnology, Beijing, China). Flow cytometry was used to assess the apoptotic cells. Apoptotic cells were gated at FSC-H and SSC-H > 100. The quantitation of apoptotic cells was calculated by CellQuest software.

### Detection of ROS by flow cytometry

Cells were suspended in diluted DCFH-DA and incubated at 37°C for 20 min. The incubated cells were washed three times with serum-free medium to remove free DCFH-DA and loaded on a flow cytometer for detection. Stained cells were gated at FSC-H and SSC-H > 150.

### Lysosome activity assay

Lysosome activity using Lyso-tracker Kit according to supplier’s instructions, and stained cells were observed under fluorescence microscopy or laser scanning confocal microscopy.

### Toluidine Blue staining

Toluidine Blue staining was performed as described [19].

### Intervertebral disc fibrosus cells and cyclic deformation treatments

The cells were isolated and cultured as described [20]. The cultured cells were subjected to low (6% deformation), medium (12 % deformation) and high (18 % deformation) cyclic (6 cycles/min) deformation treatments for 3 days using the Flexcellint Tension systems as previously described [21,22]. This system applies tension and fluid shear to generate stretch injury in cultured cells. Untreated cells were used as control.

### Transfection of siRNA lentivirus and 4-PBA treatment

Lentivirus carrying siRNAs of the CHOP gene were designed and synthesized at General Biologicals, Anhui, China. The sequences of CHOP-siRNAs are listed in Table 2. Cultured fibrosus cells were transfected with siRNA lentivirus or treated with 4-PBA (9 mM) alone or in combination before or after high cyclic deformation treatment.

**Table 2:** siRNAs used in knockdown experiments

siRNA	Sequence (5′–3′)
CHOP-siRNA-1 sense	GGUCCUGUCCUCAGAUGAAdTdT
CHOP-siRNA-1 antisense	UUCAUCUGAGGACAGGACCdTdT
CHOP-siRNA-2 sense	GAAGAUCAAGGAAGAACUAdTdT
CHOP-siRNA-2 antisense	UAGUUCUUCCUUGAUCUUCdTdT
CHOP-siRNA-3 sense	GGCUCAAGCAGGAAAUCGAdTdT
CHOP-siRNA-3 antisense	UCGAUUUCCUGCUUGAGCCdTdT
CHOP-siRNA-4 sense	ACGAAGAGGAAGAAUCAAAdTdT
CHOP-siRNA-4 antisense	UUUGAUUCUUCCUCUUCGUdTdT
CHOP-siRNA-5 sense	GAUUCCAGUCAGAGUUCUAdTdT
CHOP-siRNA-5 antisense	UAGAACUCUGACUGGAAUCdTdT
CHOP-siRNA-6 sense	GCAGGAGAAUGAGAGGAAAdTdT
CHOP-siRNA-6 antisense	UUUCCUCUCAUUCUCCUGCdTdT
FAM NC	ACGUGACACGUUCGGAGAAdTdT

### Statistical analysis

Statistical analyses were performed using GraphPad Prism 5.0 (GraphPad Software Inc., U.S.A.). All experiments were repeated at least three times and performed in triplicate. Data were expressed as means ± standard error of the mean (SEM) obtained from at least three independent experiments. Means were compared using the Student’s *t* test or one-way ANOVA with the corresponding post-test. A *P*≤0.05 was considered statistically significant.

## Results

### Intervertebral disc degeneration modeling

**Figure 1:**
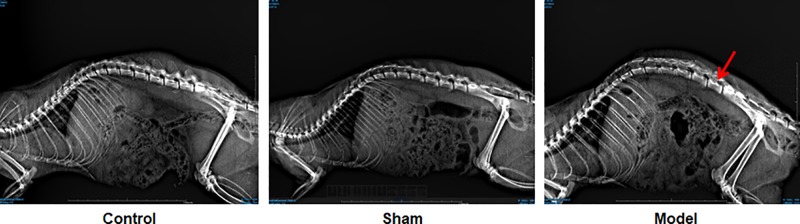
X-rays of vertebrae 3 months after modeling operation Arrow indicated narrowed intervertebral space.

Three months after modeling operation, X-rays examinations showed that rats in control and sham groups had regular intervertebral space and stable vertebrae. After the modeling operation, the animals showed narrowed intervertebral space and irregularly arranged vertebrae (Figure 1), suggesting that the modeling is successful.

### HE staining

**Figure 2:**
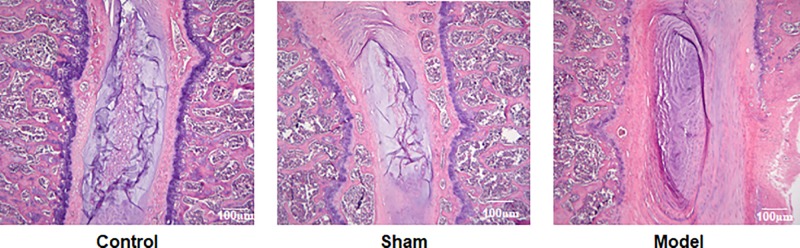
HE staining results of intervertebral disc tissue

As shown in Figure 2, for control rats, there were clear boundaries of red and light-blue staining, with complete light blue periphery in the light-blue staining areas, indicating that the cartilage endplate is intact. The intertwined red and blue linear stripes inside the light-blue staining areas were made up with fibrocartilage and collagen fibers in the nucleus pulposus. The pink-colored cartilage endplates and the bone tissue were well defined, suggesting that the structure of the intervertebral disc is intact. For rats in sham group, the staining was similar to that in control with intact cartilage endplates and nucleus pulposus. No other pathological manifestations, such as inflammatory infiltration, were observed. In the models, there were light blue ring structures with alternative blue and red edge. The loose reticular structures were not visible and the staining boundaries were unclear. The cartilage endplate and the bone tissue were not well distinguishable and the cartilage endplates were not intact, suggesting that persistent stress had resulted in the degradation in intervertebral disc and formation of cartilaginous bone.

### Apoptosis

**Figure 3:**
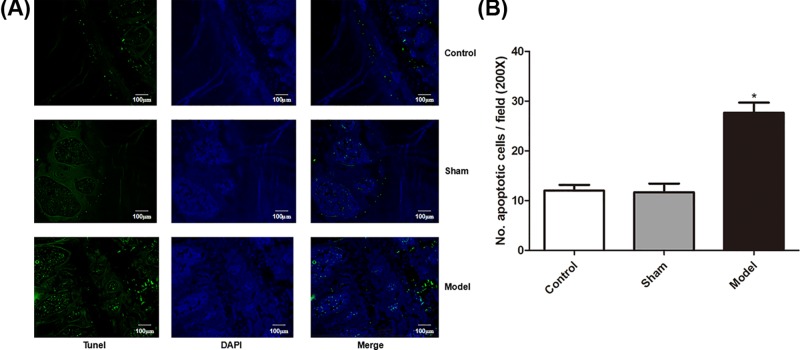
Apoptosis in intervertebral disc tissue of rat model (**A**) TUNEL assay results; (**B**) apoptotic rate. * denotes *P*<0.05 versus control.

We then examined the apoptosis in the intervertebral disc tissue. The TUNEL assays showed that there were significantly increased apoptosis in the models as compared with control and sham groups (*P*<0.05, Figure 3).

### Gene expression

**Figure 4:**
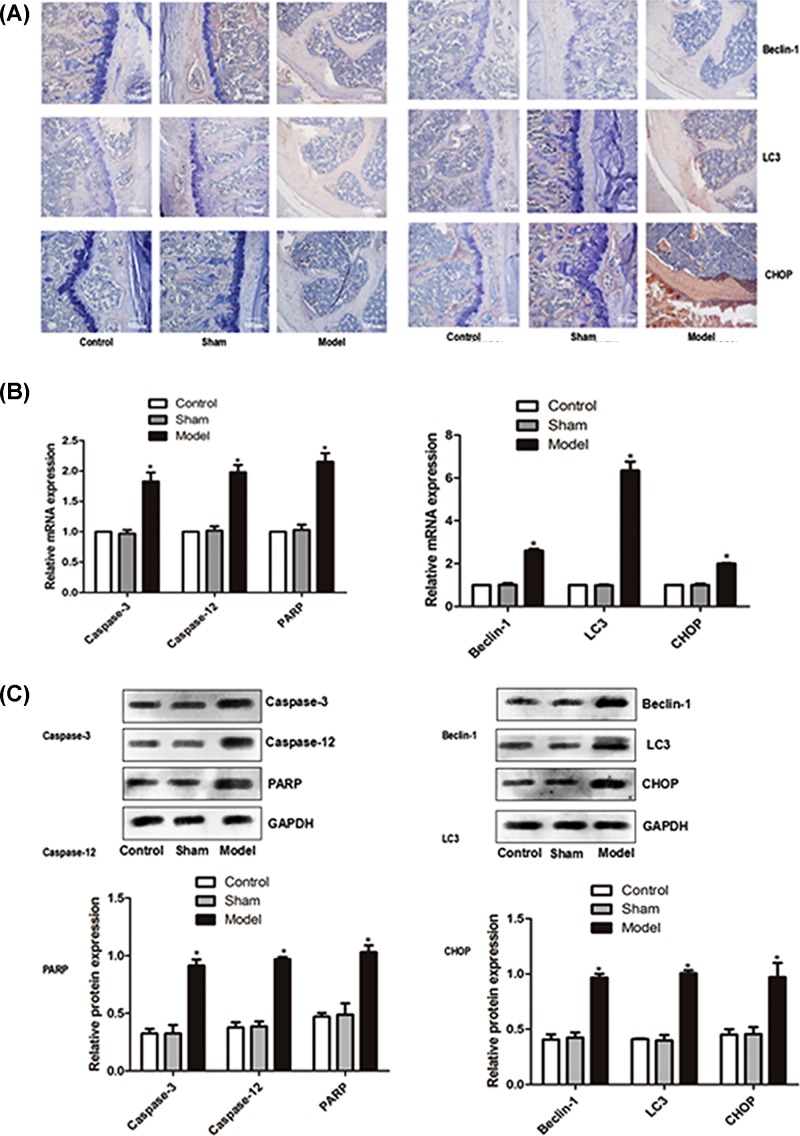
Expression of PARP, Caspase-12, Caspase-3, LC3, Beclin-1 and CHOP in intervertebral disc tissue of rat models (**A**) Immunohistochemistry result, (**B**) mRNA determined using qPCR, (**C**) Relative protein levels determined using Western blot analysis. * denotes *P*<0.05 versus control.

To figure out the molecular mechanism underlying the increased apoptosis, we determined the expression of relevant genes. Immunohistochemistry, Western blot and qPCR analyses showed that the expression of Poly(ADP-ribose) polymerase (PARP), Caspase-12, Caspase-3, LC3, Beclin-1 and CHOP was significantly increased at mRNA and protein levels in the models (*P*<0.05, Figure 4).

### Cyclic deformation stress

**Figure 5:**
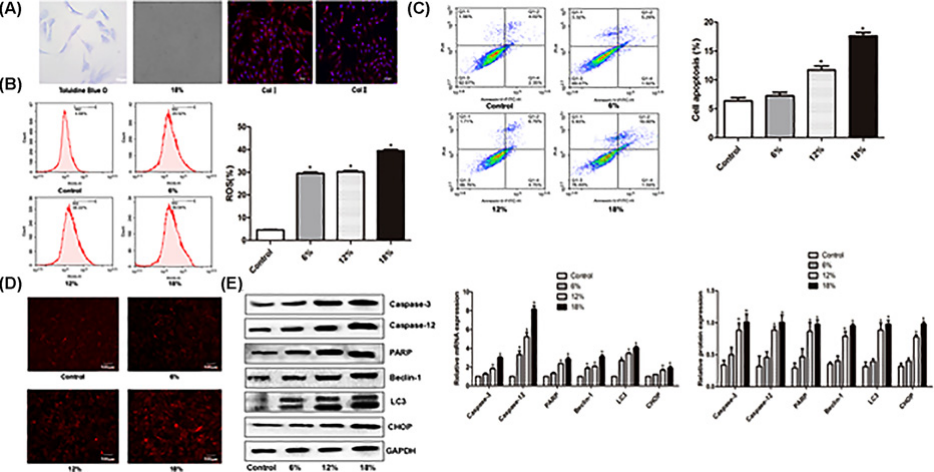
ROS production, apoptosis and expression of PARP, Caspase-12, Caspase-3, LC3, Beclin-1 and CHOP in cultured intervertebral disc annulus cells after cyclic deformation stress (**A**) Toluidine blue staining, stretching stress and immunofluorescence assays of type I and type II collagens. (**B**) Left pane: cytometry results, right pane: ROS production. (**C**) Left pane: cytometry results, right pane: apoptotic rate. (**D**) Lyso-Tracker red staining. (**E**) Left pane: representative Western blots, right pane: relative protein levels.* denotes *P*<0.05 versus control.

To mimics flexion and extension-induced tension at cellular level, we applied cyclic deformation to the cultured cells using the Flexcellint tension systems. The system introduced tension and fluid shear systems for applying mechanical load to cells in monolayer culture, which could simulate the physical stimulation of organs or tissues, providing a research platform for investigating the mechanism of structural and functional changes. The cells before deformation stress were round- or spindle-shaped and could be stained with toluidine blue (Figure 5A) and deformed under the stress ((Figure 5A). Immunofluorescence assays showed that the cells were expressing type I and type II collagens (Figure 5A). After applying the cyclic deformation, ROS and apoptosis were increased, particularly at high deformation level (18%, Figure 5B,C). After 18% deformation stress, more cells were stained red as compared with control, suggesting that the activity of lysosome is increased (Figure 5D). Meanwhile, Western blot analysis showed that the expression of PARP, Caspase-12, Caspase-3, LC3, Beclin-1 and CHOP was also increased significantly after 12 and 18% deformation stress. (*P*<0.05, Figure 5E).

### siRNA and 4-PBA treatments

**Figure 6:**
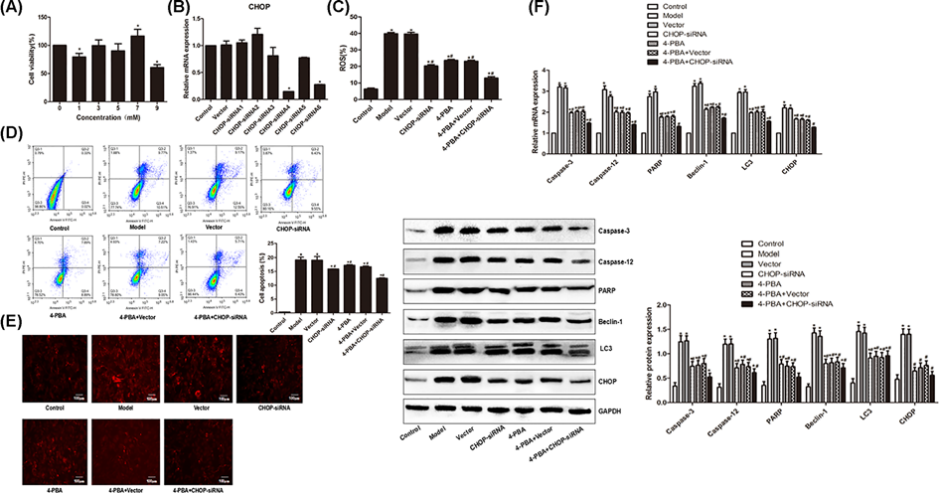
Impact of siRNA and 4-PBA treatments on cyclic deformation-induced effect in intervertebral disc annulus fibrosus cells (**A**) Cell viability after 4-PBA treatment. (**B**) Relative mRNA levels after CHOP-siRNA transfection. (**C**) ROS production after 4-PBA and CHOP-siRNA4 treatments. (**D**) * left pane: cytometry results, right pane: apoptotic rate. (**E**). Lyso-Tracker red staining. (**F**) Relative mRNA level after 4-PBA and CHOP-siRNA4 treatments. * denotes *P*<0.05 versus control.

We then investigated the impact of siRNA and 4-PBA treatments on cyclic deformation-induced effect. Pilot studies showed that optimal concentration of 4-PBA was 9 mM, which significantly reduced the viability of cells (Figure 6A), and CHOP-siRNA4 gave the greatest down-regulation of CHOP (Figure 6B). When the annulus fibrosus cells were treated with 4-PBA or CHOP-siRNA4 alone or in combination, the levels of ROS were significantly reduced (*P*<0.05, Figure 6C). Similarly, these treatments significantly reduced apoptosis (Figure 6D), lysosome activity (Figure 6E) and expression of PARP, Caspase-12, Caspase-3, LC3, Beclin-1 and CHOP (Figure 6F).

## Discussion

Intervertebral disc degeneration results from a variety of factors. Mechanical factors may induce or aggravate the process of the degeneration via reducing the nutrient supply to the intervertebral disc, therefore it is considered to be the key factor leading to the degeneration of intervertebral disc. Several approaches have been developed to construct animal models of intervertebral disc degeneration to investigate the underlying mechanism [23–25]. In the present study, we used indirect injury to the intervertebral disc to generate the models. Three months after the operation, X-rays examinations and HE pathological staining showed that the models were successfully established, where the animals had narrowed vertebral space, irregularly arranged vertebra and calcificated cartilages with collapsed nucleus pulposus, stratified and fissured annulus fibrosus.

Earlier study showed that under strong stress, blood circulation in the vertebral body may be reduced, leading to reduced number of vessels on the endplates and the malnutrition of the medullary nucleus, accelerating the process of intervertebral disc degeneration [26]. Continuous calcification and ossification of the cervical cartilage endplate may result in malnutrition in the cervical intervertebral disc, and subsequent initiation of cervical disc degeneration, as well as the aggravation of intervertebral disc degeneration [27]. At cellular level, apoptosis is shown to be closely related to lumbar intervertebral disc degeneration. For example, in mature and aged disc, apoptosis rate is significantly increased in the nucleus pulposus tissue [28]. For instance, in the rabbit models established by puncturing the lumbar intervertebral disc, the apoptosis rate increased significantly after three weeks [29]. When cells are in a state of hypoxia, oxidative damage or viral infection, proteins may not be properly folded or misfolded and calcium homeostasis may be disturbed, leading to endoplasmic reticulum stress. CHOP is an endoplasmic reticulum-related protein. When the cells are unstressed, CHOP expression is very low. Once stressed, CHOP expression is up-regulated and may result in apoptosis if the stress is excessive or persistent [30]. We find that in the model, CHOP expression as well as ROS level were significantly increased. Caspase-12 and Caspase-3 are apoptosis-related proteins, which play an irreplaceable role in apoptosis. PARP is a nucleus localized enzyme that is closely related to DNA repair under stress. It can be cleaved by Caspases *in vitro* and mainly by Caspase 3 *in vivo*. PARP is also an apoptosis marker [31]. Some studies have shown that Caspase-12 is a protease specifically activated by endoplasmic reticulum stress, which also activates Caspase 3 and other effector caspases, leading to the cleavage of PARP and other intracellular substrates and apoptosis [32]. In the rat models, PARP, Caspase-12 and Caspase-3 were all increased significantly, suggesting that the intervertebral disc degeneration may results in apoptosis. This is consistent with the above studies, indicating that endoplasmic reticulum stress is associated with apoptosis.

Autophagy is a complex process and is regulated via many signal transduction pathways. Autophagy can be induced in a variety of conditions and factors such as growth factor, amino acid level, glucose level, DNA damage and oxygen free radical [33]. Beclin1 and LC3 are the main proteins involved in autophagy. Beclin1 plays an important role in the initial phase of autophagy, which forms a trimer with P13K (h VPS34) and Atg14 that recruits continuous autophagy-related proteins to induce autophagy [34]. Under the action of Atg4, the LC3 precursor is processed to soluble LC3-I, which is linked to phosphatidyl ethanolamine (PE) to form lipid soluble LC3-II–PE under the catalysis of Atg3 (E1 like enzyme) and Atg3 (E2 like enzyme). LC3-II–PE extends the membrane of autolysosome to form autolysosome [35,36]. Studies have shown that endoplasmic reticulum stress can induce autophagy [37] and the activation of autophagy is achieved through the activation of death associated proteins that phosphorylate Beclin-l [38]. We find that autophagy-related proteins Beclin1 and LC3 were significantly increased in the model, indicating that intervertebral disc degeneration may lead to autophagy and Beclin1 and LC3 may be involved in the occurrence of autophagy as a result of endoplasmic reticulum stress.

In order to further elucidate the induction of autophagy by cyclic tension via endoplasmic reticulum stress and inhibition of oxidative stress-induced apoptosis, we subjected cultured annulus fibrosus cells isolated from intervertebral disc to deformation stress. After the stress, the cells produced increased amount of ROS and had elevated apoptosis level and lysosome activity. The expression of apoptosis-related proteins PARP, Caspase-12 and Caspase-3, endoplasmic reticulum stress-related protein CHOP and autophagy-related proteins was all up-regulated following the stress. These data further indicate that stress increases apoptosis, autophagy and level of ROS in a dose-dependent way. ROS has been shown to be the main inducer of endoplasmic reticulum stress and the main cause of autophagy [39–41]. Our earlier study indicated that autophagy in the intervertebral disc cells is mediated by ROS, and long-term nutritional deprivation may reduce the ROS content and suppress excessive autophagy to reduce the apoptosis [12]. Therefore, we speculated that stress-induced apoptosis and autophagy are mediated by ROS-dependent endoplasmic reticulum stress pathway. To verify this, we applied RNAi and endoplasmic reticulum inhibitor 4-PBA to the cultured cells. Analysis shows that both CHO-siRNA and 4-PBA alone or in combination reduced ROS production, apoptosis and expression of related genes. These results further demonstrate that stress-induced apoptosis and autophagy are mediated by ROS-dependent endoplasmic reticulum stress pathway, and inhibiting endoplasmic reticulum stress or down-regulating endoplasmic reticulum stress or down-regulating endoplasmic reticulum stress-related proteins would inhibit apoptosis and autophagy.

Taken together, the present study has demonstrated that endoplasmic reticulum stress-mediated autophagy reaction plays important role in cyclic tension induced-degeneration of intervertebral disc tissue. The degradation is an interactive results of apoptosis, autophagy and reticulum stress and is ROS-dependent.

## Availability of Data and Material

The datasets used and/or analyzed during the current study are available from the corresponding author on reasonable request.

## Abbreviations

HE: Hematoxylin and Eosin
PARP: poly(ADP-ribose) polymerase
PE: phosphatidyl ethanolamine
